# Mechano-Organ-on-Chip for Cancer Research

**DOI:** 10.3390/ijms27031330

**Published:** 2026-01-29

**Authors:** Luyang Wang, James Chung Wai Cheung, Xia Zhao, Bee Luan Khoo, Siu Hong Dexter Wong

**Affiliations:** 1 School of Medicine and Pharmacy, Ocean University of China, Qingdao 266003, Chinazhaoxia@ouc.edu.cn (X.Z.); 2 Laboratory for Marine Drugs and Bioproducts, Qingdao Marine Science and Technology Center, Qingdao 266237, China; 3 Department of Biomedical Engineering, Faculty of Engineering, The Hong Kong Polytechnic University, Kowloon, Hong Kong SAR, China; james.chungwai.cheung@polyu.edu.hk; 4 Department of Biomedical Engineering, College of Biomedicine, City University of Hong Kong, Kowloon, Hong Kong SAR, China; 5 Hong Kong Centre of Cerebro-Cardiovascular Health Engineering (COCHE), Shatin, Hong Kong SAR, China

**Keywords:** Mechano-Organ-on-Chip, tumor microenvironment mechanics, microphysiological systems, mechanotransduction

## Abstract

Mechano-Organ-on-Chip (Mechano-OoC) platforms are emerging as powerful microphysiological systems that place mechanical cues at the center of tumor modeling, providing a scalable and human-relevant approach to recapitulate the biophysical complexity of the tumor microenvironment. Mechanical factors such as matrix stiffness, viscoelasticity, solid stress, interstitial flow, confinement, and shear critically regulate cancer progression, metastasis, immune interactions, and treatment response, yet remain poorly captured by conventional in vitro models and are often studied separately in tumor-on-chip and mechanobiology research. In this review, we summarize recent advances in mechano-OoC technologies for cancer research, highlighting strategies that integrate engineered mechanical cues with microfluidics, tunable extracellular matrices, vascular and stromal interfaces, and dynamic loading to model tumor invasion, vascular transport, immune trafficking, and drug delivery. We also discuss emerging approaches for real-time, multimodal readouts, including sensor-integrated platforms and artificial intelligence-assisted data analysis, and outline key challenges that limit translation, such as device complexity, limited throughput, insufficient standardization, and inadequate validation against in vivo and clinical data. By organizing progress across platform engineering, sensing and readout, data standardization, and AI-driven analytics, this review provides a unified framework for advancing mechanobiology-aware tumor models and guiding the development of predictive preclinical platforms for precision cancer therapy.

## 1. Introduction

It is increasingly recognized that the tumor microenvironment (TME) is governed by not only biochemical factors but also by physical and mechanical cues. Examples of such mechanical factors include extracellular matrix (ECM) stiffness, compressive solid stress, interstitial fluid pressure/flow, geometric confinement, and shear forces [1,2,3]. These forces influence key cancer phenotypes, including cell proliferation, survival, motility/invasion, epithelial–mesenchymal transition (EMT), stemness, and drug resistance [4]. Mechanotransduction, which is the process by which cancer cells sense these mechanical signals via the cytoskeleton, adhesion complexes, and nuclear deformation, can rewire gene expression and alter cellular behavior [5]. Moreover, non-malignant TME components and the ECM itself respond to such mechanical cues, affecting tumor progression, metastasis, immune interactions, and drug delivery [6,7]. Collectively, we emphasize that faithful tumor models must reproduce both the biochemical and mechanical dimensions of the TME.

Conventional preclinical models have major limitations in recapitulating tumor mechanics. Two-dimensional cell cultures entirely lack three-dimensional architecture, native ECM, fluid flow, and relevant mechanical forces [8]. Even advanced three-dimensional models, such as multicellular spheroids or organoids, which capture some cell–cell interactions, generally lack perfused vasculature, interstitial shear stress, stromal and immune components, and fail to reproduce the mechanical stresses experienced in vivo [9,10,11,12]. Animal models incorporate physiological complexity but introduce species-specific differences in ECM composition and immune context, incur high cost and ethical concerns, and limit high-throughput experimentation and real-time monitoring [13]. Consequently, there is a critical need for scalable, human-relevant in vitro platforms that integrate biochemical, cellular, and mechanical complexity [14,15,16]. We therefore propose that mechanobiology-aware Organ-on-Chip (Mechano-OoC) systems, which combine microfluidics, tissue engineering, and controlled mechanical cues, offer a promising solution to bridge this gap.

In this review, we highlight how OoC technology leverages microfabrication, microfluidics, and engineered biomaterials to create microphysiological systems that recapitulate key organ-level functions, incorporating controlled fluid flow, mechanical forces, multiple tissue interfaces, and three-dimensional architecture with human cells [17,18,19]. Recent studies emphasize that microfluidic OoC models can recreate tumor-like microenvironments while enabling precise tuning of biochemical and biophysical parameters. Given the critical role of mechanical forces in driving cancer invasion, metastasis, drug transport, and immune interactions, we focus on the growing need to integrate mechanobiology into OoC platforms; mechano-OoC systems, by incorporating engineered mechanical cues into tumor-on-chip models, offer a promising solution to achieve more realistic replication of these processes [20,21,22]. We summarize recent advances in engineering these platforms (including tunable ECM mechanics, dynamic loading, and multicellular interfaces), multimodal sensing and real-time readouts (with sensor integration), data standardization, and AI-driven image analysis and predictive modeling, while addressing challenges in scalability, reproducibility, and clinical validation to advance mechano-OoC as predictive tools for precision cancer therapy [23,24,25].

## 2. Engineering Mechano-OoC Platforms: Recapitulating the Mechanical Tumor Microenvironment

### 2.1. Key Mechanical Dimensions in the Tumor Microenvironment

Several key mechanical features characterize solid tumors. A central factor is the extracellular matrix: many tumors exhibit increased ECM cross-linking, fiber alignment, and density, yielding higher stiffness [26,27,28]. Changes in matrix viscoelasticity also modulate cell mechanosensing and behavior [29,30]. Another hallmark is growth-induced solid stress: as tumors expand and deposit ECM, compressive stresses build up that physically squeeze adjacent tissues and vessels, altering interstitial pressure and perfusion [31,32]. The geometry of the ECM, which includes features like pore size, fiber orientation, and physical confinement, further influences how cells migrate and how their nuclei deform under stress [33,34]. Importantly, the TME is heterogeneous and dynamically remodels over time: for example, matrix stiffness and architecture change through ECM deposition, degradation, and cell-driven remodeling [35,36,37]. These mechanical factors interact (e.g., ECM stiffness affects stress distribution, and fluid flow depends on matrix porosity), suggesting that mechano-OoC platforms should incorporate multiple cues simultaneously rather than focusing on a single parameter [38,39].

The competitive advantage of mechano-OoC platforms is not merely the ability to present multiple mechanical cues, but the capacity of microfluidic engineering to decouple those cues so they can be varied independently and quantitatively. Classic multi-layer designs use isolated vacuum or pneumatic drive channels that deform a thin membrane or hydrogel layer to apply cyclic stretch while keeping perfusion streams separate, enabling stretch amplitudes and frequencies to be changed without altering channel flow fields [40]. This architecture was pioneered in lung-on-a-chip devices and remains a canonical example of independent actuation.

Likewise, hydrogels or 3D matrices can be sandwiched or spatially confined in dedicated gel chambers that are fluidically isolated from adjacent perfusion channels. By imposing a controlled pressure difference across the gel or by perfusing parallel side channels, researchers can generate interstitial flow through the matrix while maintaining a low shear stress on cells at the matrix–channel interface [41]. Such geometries permit independent modulation of interstitial velocity and luminal shear stress. Beyond static geometry, tunable materials and active hydrogel strategies offer another route to decoupling: phototunable or chemically triggered hydrogels and actuatable hydrogel membranes allow in-platform changes in local matrix stiffness without changing external flow conditions, so stiffness-dependent mechanotransduction can be probed while holding convective transport constant [42].

### 2.2. Mechano-OoC Design Principles and Material Selection

General guidelines for Organ-on-Chip design have been articulated in recent research. To develop OoC systems, it is essential to start with a strong baseline that encompasses critical elements such as suitable materials, standardized cell seeding approaches, regulated microfluidic flow, and reliable readout strategies [43,44]. Extending these principles to cancer models involves adding modules for mechanical control. The same microfabrication and fluidic toolkits can be used, but the design must explicitly incorporate tunable ECM mechanics, on-chip force application, integrated sensors, and means for dynamic perturbation. Using collagen-gelatin composite hydrogels (with a tunable stiffness of 1–10 kPa) enables the accurate reproduction of the high-stiffness microenvironment of the pancreatic cancer stroma, thereby providing a controllable model for studying the matrix stiffness-induced EMT process [39,45].

A major technical limitation in tumor-on-chip platforms for drug screening lies in the non-specific adsorption and absorption of hydrophobic small-molecule drugs by polydimethylsiloxane (PDMS). Owing to its hydrophobic and porous polymer network, PDMS can significantly deplete the free concentration of many chemotherapeutic agents from the perfusion medium in a time- and compound-dependent manner [46]. This effect is particularly problematic in mechano-OoC systems, where long-term perfusion and cyclic mechanical stimulation can exacerbate drug partitioning into the PDMS bulk, thereby distorting the intended dose, exposure kinetics, and reproducibility of pharmacological readouts. Consequently, the apparent cellular response may reflect material–drug interactions rather than true mechano-pharmacological effects.

To mitigate these issues, surface coatings or PDMS pre-saturation strategies have been explored, yet these approaches often provide incomplete or unstable suppression of sorption and may compromise gas permeability or mechanical compliance under repeated deformation. As a result, alternative materials are increasingly adopted for quantitative drug screening applications. Thermoplastic polymers such as polystyrene (PS), poly methyl methacrylate (PMMA), and cyclic olefin copolymer/polymer (COC/COP) exhibit substantially reduced small-molecule sorption and improved chemical fidelity [47]. However, their limited elasticity necessitates hybrid or composite device designs, in which rigid thermoplastic microfluidic layers are combined with thin elastomeric or thermoplastic elastomer membranes to preserve mechanical actuation. Material selection thus represents a critical design parameter in mechano-OoC systems, particularly when accurate drug concentration control under dynamic mechanical loading is required for translational relevance.

### 2.3. Existing Mechano-OoC Platform Implementations and Applications

**Figure 1:**
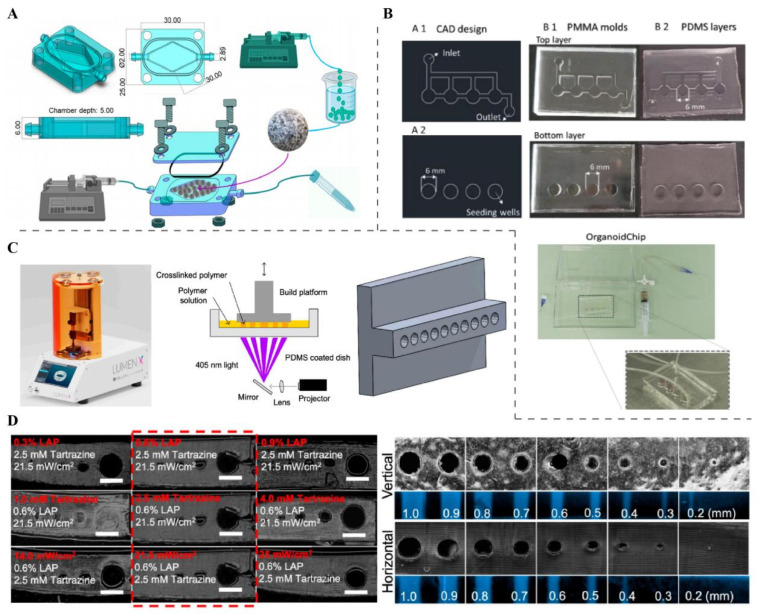
**Design and fabrication of the mechano-OoC platforms.** (**A**) Colorectal tumor-on-chip design and configuration comprises a SolidWorks-designed (SolidWorks 2024) 3D-printed mini-reactor with defined shape and dimensions, Caco2 microtumors in hydrogel microspheres fabricated by dripping bioink (3.5% alginate, 2.5% GelMA, 1.5 × 10^6^ cells/mL) into 4% CaCl_2_ and 12 g/L Tween 80 bath, and a closed system via acrylic lid sealing and connection to syringe pump and waste tube [48]. (**B**) The design and fabrication of the organoid-on-chip device involve AutoCAD-designed top (AutoCAD v25.1) (one inlet channel splitting into four sub-channels and one outlet) and bottom (four 6-mm-diameter round wells for organoid seeding/growth) layers, PMMA molds fabricated via milling machine for producing corresponding PDMS layers, and subsequent device setup [51]. (**C**) The figure includes an image of the LumenX DLP printer (**left**), a schematic of a basic DLP printer in operation (**middle**), and a CAD model for printing vertically oriented channels of varying sizes (**right**) [53]. (**D**) Printable PEGDA channel diameter varies with single-component adjustments (LAP/tartrazine concentration, projector power); microscope images of 0.1–1.0 mm channels and fluorescent dye confirm hollowness [53].

Several recent publications illustrate the promise of tumor-on-chip systems in cancer research. These current studies aim to establish a platform that can reproduce the significant characteristics of tumors, including three-dimensional structure, natural extracellular matrix, perfusion, heterogeneity, and even angiogenesis, for the purposes of cancer biology, metastasis, and drug testing studies (Figure 1A) [48,49]. More recent experiments have co-cultured tumor cells with stromal cells in microfluidic chips, modeling aspects of solid tumor growth under well-controlled microenvironmental conditions (Figure 1B) [50,51]. Similarly, emerging efforts combine tumor organoids with microfluidics to create more physiologically relevant models. By integrating mini-tumors or organoids on a chip, researchers better mimic tumor heterogeneity and ECM context while enabling drug response studies in a dynamic environment (Figure 1C,D) [52,53]. Collectively, these works provide proof-of-concept that tumor-on-chip platforms, which, by extension, include mechano-OoC systems, are both feasible and promising for cancer research. While many tumor-on-chip studies focus on reconstructing tissue architecture and establishing perfusion, integrating explicit mechanical cues (e.g., matrix stiffness gradients or cyclic compression) remains challenging. To address matrix mechanics, researchers employ tunable hydrogels or biomaterial scaffolds (such as collagen, fibrin, gelatin, or synthetic polymers) whose properties (stiffness, viscoelasticity, porosity, crosslinking) can be varied [54,55]. Embedding cancer cells or tumor organoids in such matrices can recreate the stiffer, desmoplastic ECM often seen in tumors [56,57]. At the same time, microfluidic platforms naturally allow for controlled flow and perfusion: by tuning channel geometry, flow rates, and matrix permeability, one can impose interstitial flow, generate fluid shear, and create gradients of nutrients or drugs [20,58]. This approach is well-established in physiological organ chips (e.g., vascular or renal models) and can be adapted to tumor chips to study transport phenomena [59].

Chip designs that include endothelialized vasculature alongside stromal and tumor compartments allow detailed modeling of blood-tumor interactions. For instance, chips incorporating endothelial-lined channels adjacent to tumor or stromal compartments can replicate angiogenesis, immune cell trafficking, and drug transport across the vasculature. Such vascularized multi-tissue chips have been demonstrated in other OoC contexts, suggesting that tumor-on-chip efforts can leverage similar strategies to mimic angiogenic remodeling and metastatic intravasation. Including multiple cell types on-chip further enables the study of complex immune and stromal interactions under flow [60,61]. So far, few high-impact studies have explicitly applied dynamic mechanical stress (cyclic strain, compression) to tumor-on-chip systems [62]. This reflects a critical challenge: to date, mechano-OoC platforms that subject tumour tissues to engineered cyclic deformation or solid stress are still relatively rare [63]. In summary, while OoC technologies are mature for replicating tissue architecture, perfusion, and cellular heterogeneity, truly mechanobiology-aware OoC systems, which incorporate controlled mechanical stress and dynamic stimuli, remain largely experimental [14,64]. We argue that overcoming these hurdles is essential to harness the full potential of mechano-OoC in cancer research.

Dynamic mechanical loading applied externally should be clearly distinguished from solid stress generated endogenously by tumor growth. Solid stress arises from volumetric expansion of proliferating tumor cells within a mechanically confining microenvironment and results in sustained, inward-directed compressive forces with biological effects distinct from transient external strain. In tumor-on-chip platforms, such growth-induced solid stress can be modeled and quantified by exploiting microstructural confinement, for example by embedding tumor spheroids within mechanically defined hydrogel chambers or microfabricated cavities [65]. As spheroids expand, deformation of the surrounding matrix or integrated elastic microstructures enables estimation of solid stress [66]. Moreover, chip-level physical confinement—through controlled chamber geometry and wall or matrix stiffness—can be used to mimic the inward pressure exerted by the tumor capsule in vivo, providing a complementary mechanical modality to cyclic strain–based mechano-OoC designs.

### 2.4. Challenges, Standardization, and Path Toward Reproducible Mechano-OoC

For mechano-OoC to become widely applicable in research and preclinical settings, several challenges must be addressed. First, design parameters and reporting must be standardized. In other words, materials, flow conditions, geometry, and cell composition should be documented in sufficient detail to allow reproducibility across laboratories [67,68]. Without consistent metadata and reporting standards, it is difficult to compare results or replicate findings. Thus, the field must adopt clear guidelines so that mechano-OoC experiments can be faithfully reproduced and interpreted.

Second, many mechano-OoC devices are technically complex and have low throughput. Incorporating features like vascularization, dynamic strain, and multiple compartments often yields intricate chips that are labor-intensive to fabricate and operate. This complexity limits scalability: for applications like drug screening, a balance between physiological relevance and throughput is needed, but such trade-offs remain a major bottleneck [69]. Similarly, increasing the number of cell types (e.g., adding stromal, immune, and endothelial cells) and biomechanical stimuli enhances biological realism, but this also further complicates device design and data analysis. In short, controlling complexity while maintaining experimental control is a persistent challenge [70,71].

Third, capturing the effects of mechanical cues requires suitable sensors and readout modalities. Many current tumor-on-chip studies rely on endpoint assays or simple microscopy, but to observe dynamic processes under mechanical stimulation, real-time and high-dimensional readouts are needed [72,73]. On-chip sensors for measuring variables like oxygen, pH, interstitial pressure, or cell-generated forces would greatly enhance the ability to monitor mechano-OoC experiments in situ. Finally, validation remains a hurdle: while OoC models can emulate many in vivo features, mechano-OoC systems have seldom been benchmarked against clinical data or patient-derived models. Without such validation, the translational relevance of mechano-OoC findings remains uncertain. For example, comparing invasion or drug response in the chip to animal or patient data is crucial to confirm that the mechanical modulations produce physiologically meaningful effects [74,75,76].

In summary, although the engineering toolbox largely exists and tumor-on-chip platforms have shown promise, truly standardized and validated mechano-OoC systems remain rare. High-impact publications that integrate controlled mechanical cues with robust validation are still scarce. We argue that addressing these issues, and in particular standardization, scalability, real-time sensing, and cross-validation, will be essential for mechano-OoC to fulfill its promise in cancer research. Challenges facing mechano-OoC, such as standardization and scalability, depend not only on the engineering optimization of platforms but also require matching targeted detection technologies and data interpretation systems. Thus, the following sections will focus on strategies for sensing, detection, and data standardization.

## 3. Sensing, Readout, and Data Considerations: Toward Mechano-OoC as a Research Standard

### 3.1. Lessons from General OoC for Assays and Readouts

Engineering a mechano-OoC is only half the battle; equally important is how we measure and analyze the biological responses. Lessons from general OoC development emphasize that readouts must be tailored for microfluidic systems. Conventional methods, such as microscopy (phase-contrast, fluorescence), live/dead assays, and standard immunostaining, can be applied. Still, work focusing on OoCs highlights the importance of compatibility with on-chip culture, such as optical access and non-destructive sampling. More advanced functional assays, which include barrier permeability tests, trans-endothelial electrical resistance, and tracer-based flow analysis, are recommended to probe dynamic behaviors [77,78,79]. In cancer models, these approaches should be combined with tumor-specific readouts (e.g., invasion/migration assays, proliferation markers, hypoxia sensors, drug response assays) to capture clinically relevant endpoints [80,81]. We also note that static endpoint measurements are often insufficient in mechanobiology contexts: they can miss how cells adapt over time to changing forces or remodel the ECM [82]. Therefore, real-time, longitudinal, and multimodal readouts are necessary to exploit mechano-OoC systems fully [83].

### 3.2. Emerging Readout Modalities Relevant for Mechano-OoC

New readout modalities are emerging for mechano-optical coherence tomography. Because chips are often made from transparent materials, live-cell imaging techniques (such as phase-contrast, fluorescence, and confocal microscopy) can be employed to track cell morphology, migration, and interactions under flow conditions. Indeed, several tumor-on-chip studies have used time-lapse microscopy to monitor tumor growth, organoid dynamics, and drug responses in perfused environments. Time-lapse microscopy can not only monitor tumor growth, but also quantify mechanotransduction-related phenotypes including the migration rate and nuclear deformation degree of tumor cells under mechanical stimulation [50]. Barrier integrity and permeability assays are particularly relevant for vascularized chips. In microfluidic devices with an endothelial barrier, one can measure transendothelial electrical resistance or track the diffusion of fluorescent tracers to quantify barrier function under flow [84,85]. These approaches, recommended in foundational OoC research, are valuable for modeling metastasis (intravasation/extravasation) and drug delivery across vessel walls [86]. 

**Figure 2:**
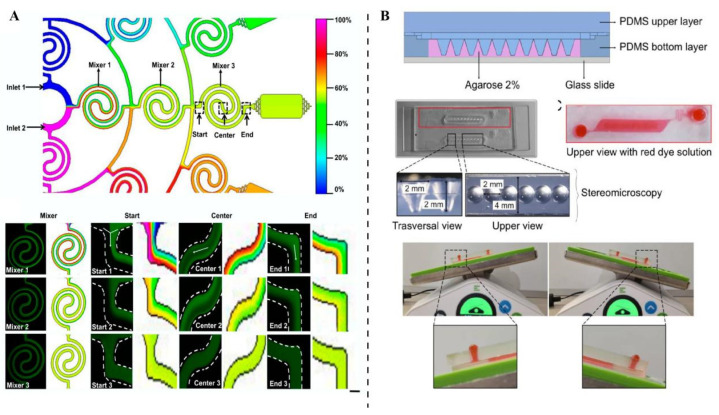
**The design of microfluidic perfusion chips and their application in drug screening.** (**A**) Microfluidic device mixing effect (20 μL min^−1^, successive spiral mixer regulation) involves device simulation imaging, with three areas (start, center, end) in three mixers for mixing status identification [87]. (**B**) Development and characterization of a spheroid-containing microfluidic platform involve PDMS-glass-agarose (cone-shaped wells) structure, assembled device images, and dye/OrganoFlow^®^ validation of no leakage and normal fluid flow [93].

Microfluidic perfusion itself offers a wealth of experimental possibilities (Figure 2A) [87]. By generating controlled gradients of nutrients, oxygen, or drugs across the chip, researchers can study how flow and ECM properties together influence drug penetration and efficacy [88,89,90]. For example, several tumor-on-chip systems have leveraged perfused microfluidic flows to perform drug screening under physiologically relevant transport conditions (Figure 2B) [91,92,93]. Mechano-OoC platforms can also integrate multiple cell types on-chip, and co-culturing tumor cells with stromal fibroblasts, endothelial cells, or immune cells in a perfused microenvironment allows studying complex processes like immune infiltration, stromal remodeling, angiogenesis, and therapy response under mechanical cues [94,95]. Although such sophisticated multi-cell chips are more common in non-cancer organ-chip models, the same design principles, such as multi-cell co-culture under perfused mechanical microenvironments, apply to tumor-on-chip systems [96,97].

Most tumor-on-chip platforms rely on pre-patterned, endothelial-lined microchannels to model perfusion and drug delivery; however, such architectures fail to recapitulate the structural irregularity and leakage characteristic of tumor angiogenesis. In contrast, self-assembled microvascular networks formed via endothelial sprouting more closely reproduce heterogeneous vessel diameters, disrupted junctions, and localized leakage, which are key contributors to elevated tumor interstitial fluid pressure [98]. Because vascular permeability directly governs interstitial pressure buildup and convective drug transport, tumor chips that lack physiologically relevant vascular leakage may substantially underestimate transport barriers and drug exposure gradients. Incorporating self-assembled vasculature therefore provides a more faithful mechanical and transport microenvironment for modeling tumor interstitial pressure and drug delivery than idealized pre-patterned channels. Finally, there is great potential to integrate on-chip sensors. For instance, embedded microsensors could continuously monitor oxygen tension, pH, interstitial pressure, matrix deformation, or cell-generated traction forces within the chip [99,100]. While mechano-OoC studies with such integrated sensors are still rare, developing these capabilities should be a priority in future work [101,102].

### 3.3. Sensor-Integrated and AI-Enhanced Readouts for Mechano-OoC

Recent progress has moved well beyond conventional imaging and trans-epithelial/endothelial electrical resistance (TEER), introducing sensor-integrated platforms that provide continuous, multi-dimensional monitoring of mechanical and biochemical signals inside Organ-on-Chip systems [103]. These technologies address a critical gap by enabling real-time mechanobiology analysis and improving translational relevance, particularly in dynamic microenvironments where cyclic strain, shear stress, and perfusion gradients shape cellular behavior. Conventional imaging and endpoint assays remain useful, but they cannot capture the complex temporal patterns of mechanotransduction [104,105,106]. To overcome this limitation, researchers are embedding advanced sensors directly into chip architectures and pairing them with AI-driven analytics, a shift that transforms Organ-on-Chip models into adaptive, high-content systems capable of delivering mechanistic insights with unprecedented precision [107]. 

**Figure 3:**
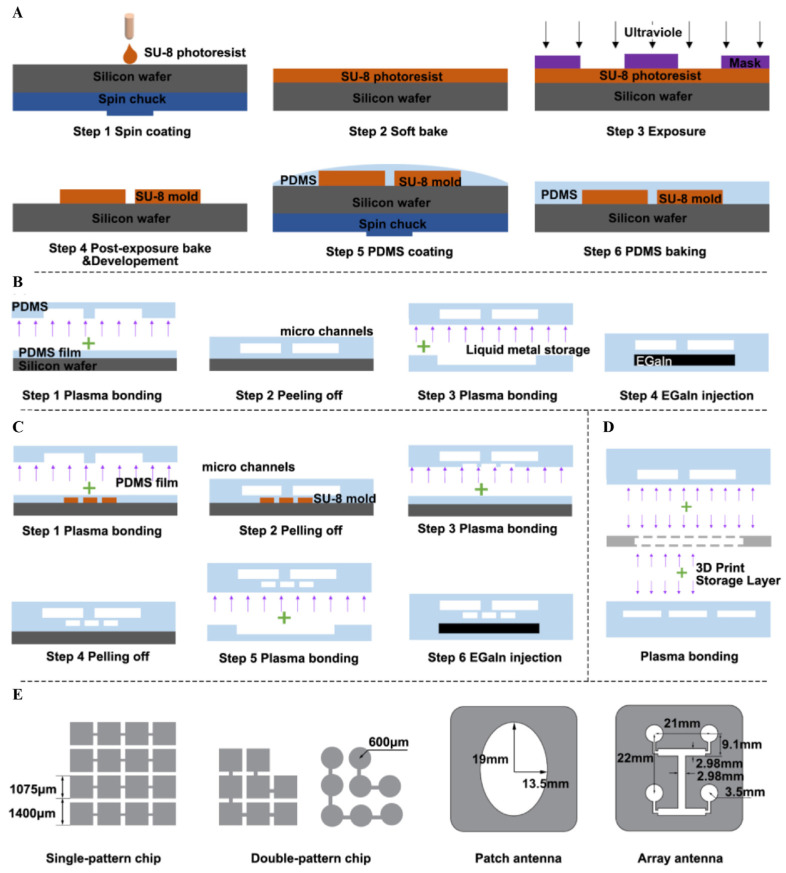
**Fabrication processes and dimensional details relevant to the pressure-driven rapid reconfigurable liquid metal patterning strategy.** (**A**) Standard soft lithography for PDMS component fabrication, (**B**,**C**) preparation of single- and double-pattern chips, (**D**) preparation of dual-frequency reconfigurable antennas, (**E**) and specific dimensional parameters of liquid metal patterns formed by elastic polymer film deformation in the “pattern—film—cavity” sandwich structure under working medium pressure [108].

One important advance is the creation of moisture-permeable, deformable circuits that remain electrically stable under mechanical strain and fluid exposure. Designs such as liquid-diode electronics enable the direct integration of sensors into microfluidic chips without compromising biocompatibility or flow performance in Figure 3 [108]. These embedded systems can continuously monitor pressure, strain, and barrier integrity during physiologic perfusion, supporting long-term studies of tissue mechanics and drug transport. Incorporating these electronics into chip substrates enables real-time mapping of stress distribution, ECM deformation, and interstitial pressure, reducing the need for invasive probes. This approach also supports multiplexed sensing, allowing oxygen tension, pH, and TEER to be tracked alongside mechanical cues [109]. The combination of soft electronics and microfluidics offers a practical route toward clinically relevant Organ-on-Chip platforms, bridging the gap between early prototypes and regulatory-grade models.

### 3.4. Liquid Metal Flexible Sensors Empower Microfluidic Models 

**Figure 4:**
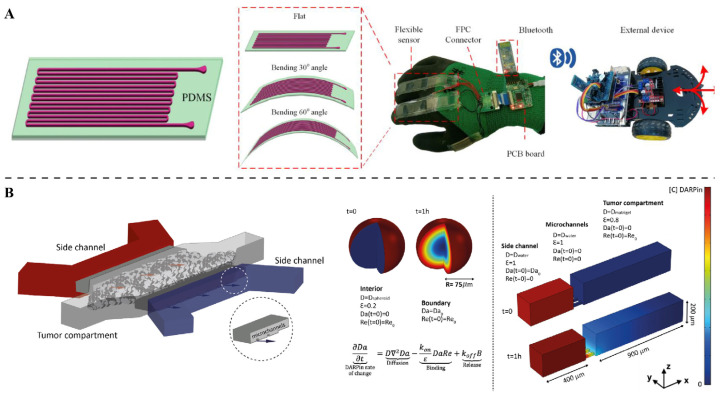
**Mechano-OoC relies on two core components: sensor-integrated AI-enhanced readouts, and data standardization, metadata, along with the path to reproducibility and translation.** (**A**) The liquid metal flexible sensor, fabricated via soft lithography, exhibits stable performance in reciprocating bending tests with a 6 mm bending radius, facilitates accurate joint bending angle detection, and realizes wireless Bluetooth car control after integration into a wearable glove [111]. (**B**) The tumor-on-a-chip system overview and mathematical model conditions for spheroids and on-chip scenarios involve a microfluidic system with side channels delivering DARPins to a tumor channel, a COMSOL-simulated spheroid model and a 3D on-chip geometry in COMSOL (COMSOL v6.3) accounting for distinct diffusion coefficients and void fractions in the tumor compartment and side channels [119].

Wearable sensors such as pressure devices and pulse monitors provide exceptional sensitivity to subtle mechanical changes (Figure 4A) [110,111]. Originally developed for cardiovascular monitoring, these sensors detect micro-scale pressure fluctuations and dynamic strain patterns; capabilities that translate effectively to mechano-OoC systems. When adapted for chip integration, they enable precise measurement of interstitial pressure, perfusion dynamics, and matrix deformation under controlled mechanical stimuli. Coupling these sensors with machine learning algorithms enhances interpretability, allowing automated classification of mechanical signatures linked to pathological states such as tumor stiffening or endothelial barrier failure [112]. In Organ-on-Chip contexts, this integration enables the real-time prediction of tissue responses under cyclic strain or shear, supporting adaptive drug dosing and mechanobiology-informed therapy design. AI-assisted sensing transforms OoC platforms from static observational models into dynamic, predictive systems.

Beyond sensor integration, recent efforts have shifted toward microfluidic 3D disease models, such as bladder cancer chips that incorporate bacterial-targeted aggregation-induced emission (AIE) photosensitizers for combined photodynamic and chemotherapeutic treatment [113]. This approach highlights how mechanical transport and barrier properties can significantly influence therapeutic outcomes, since drug penetration and light delivery depend on perfusion rates and ECM stiffness [114,115]. Adding sensors to monitor oxygen gradients, pH changes, and interstitial pressure enables these platforms to adjust treatment conditions dynamically, improving precision and efficacy. Introducing microbial components adds further complexity, both mechanical and biochemical, mimicking infection-driven inflammation, which is increasingly recognized as a factor in cancer progression [116]. These hybrid systems represent the next generation of mechano-OoC platforms: multimodal, sensor-rich, and clinically relevant, capable of revealing how mechanical and microbial cues interact to shape tumor biology and drug response. The multimodal Mechano-OoC platforms that integrate photomedicine and microbial cues generate multidimensional data, which require unified standardized procedures in particular to ensure reproducibility. Therefore, there is an urgent need to establish clear data reporting standards.

### 3.5. Data Standardization, Metadata, and Path Toward Reproducibility & Translation

To promote the wide adoption of mechano-OoC, researchers must accompany data generation with careful metadata reporting, standardization, and data-sharing (Figure 4B) [117,118,119]. Researchers should report all mechanical parameters in detail: ECM composition and stiffness, viscoelasticity, matrix architecture (fiber density, orientation), gel crosslinking, flow rates and shear stresses, pressure gradients, imposed deformation schedules (strain amplitude/frequency, compression cycles), device geometry and confinement dimensions, cell types and densities, culture conditions, and so on [102,120,121,122]. Likewise, for each readout, one should document the imaging modality (magnification, resolution, and channels), time-lapse schedule, tracer concentrations, assay protocols, and data analysis method [123,124,125].

We also advocate for sharing raw data and using standard file formats (e.g., raw imaging data, metadata in JSON/CSV, plate maps, time logs) [126]. This practice will enhance reproducibility, allow cross-study comparisons, and eventually enable pooled data sets for computational and machine-learning analyses [127]. Importantly, mechano-OoC results should be validated against in vivo or clinical data whenever possible. Comparing outcomes, such as invasion, metastasis, drug response, vascular permeability, or immune infiltration, between chip models and animal or patient-derived models is key to demonstrating translational relevance [128]. Only with such rigorous standards and validation can mechano-OoC approaches fulfill their potential as predictive preclinical platforms.

## 4. Artificial Intelligence in Organ-on-Chip: From Image Analysis to Predictive Modeling

### 4.1. AI-Driven Image Analysis for Non-Destructive Evaluation in OoC Systems

Image-based analysis is the most mature and widely used entry point of artificial intelligence (AI) in Organ-on-Chip research. OoC platforms are inherently compatible with live imaging techniques, including bright-field, fluorescence, and confocal microscopy, enabling continuous monitoring of biological processes under physiologically relevant conditions [129,130,131]. However, extracting quantitative and reproducible information from such live imaging data remains challenging when relying on manual or conventional image-processing methods.

Furthermore, deep learning models, particularly convolutional neural networks (CNNs) are increasingly employed to analyze live imaging data generated by OoC systems [132]. These models enable automated evaluation of cellular morphology, tissue organization, and dynamic structural changes without the need for invasive measurements or destructive endpoint assays [131]. By learning complex spatial features directly from raw images, CNN-based approaches can accurately segment cells and tissues, quantify morphological heterogeneity, and track structural evolution over time [133,134]. Importantly, AI-driven image analysis supports the assessment of functional properties such as barrier integrity and tissue maturation in a non-destructive manner [135]. For example, CNNs can infer barrier formation, disruption, or remodeling by analyzing spatiotemporal patterns in fluorescence or phase-contrast images, reducing reliance on invasive permeability assays or labeling-based measurements. For the mechanical simulation scenarios of mechano-OoC, AI can automatically identify the correlation patterns between matrix stiffness and cell traction force through label-free phase contrast microscopy images, and quantify the efficiency of mechanotransduction without relying on fluorescent labeling. In addition, CNNs can not only automatically segment tumor cells, but also quantify key mechanotransduction phenotypes such as cell morphological heterogeneity induced by mechanical stimuli such as matrix stiffness gradients, including long axis to short axis ratio, nuclear deformation degree, thus avoiding the subjectivity of traditional manual analysis. This capability is particularly valuable for long-term OoC experiments, where repeated destructive measurements would otherwise compromise system stability.

Overall, the application of deep learning-based image analysis enhances the ability of OoC platforms to serve as continuous, non-invasive probes of living biological systems [132,133]. By enabling real-time, label-free, and longitudinal quantification of cellular and tissue-level features, AI tools significantly expand the experimental scope of OoC research and lay the foundation for subsequent multimodal integration and predictive modeling. This technology, AI image analysis, provides mechano-OoC with a solution for high-throughput quantification of mechanical phenotypes, resolving the key challenge that traditional methods are unable to monitor the temporal relationship between dynamic mechanical stimuli and cellular responses in real time.

### 4.2. From Multimodal Feature Extraction to Predictive Modeling in OoC Systems

The core requirement of mechano-OoC is to uncover the complex correlations among mechanical cues, biochemical signals, and cellular behaviors. AI multimodal predictive modeling is exactly able to capture such nonlinear interactions, and its core value lies in transforming mechanical mechanisms that cannot be directly measured into predictable clinical outcomes. While AI-driven image analysis provides a powerful and non-destructive means to quantify biological phenotypes in OoC systems, predictive modeling in this context should not rely solely on image-derived information. Instead, robust prediction typically emerges from the integration of multiple feature types, including image-based features, manually engineered descriptors, and system-level experimental parameters [136,137]. In OoC platforms, deep learning-based image analysis yields high-dimensional representations of cellular morphology, tissue organization, growth dynamics, and spatiotemporal behavior [136]. These image-derived features capture rich phenotypic information and often serve as primary inputs for predictive models. However, many biologically and experimentally relevant variables—such as flow rates, drug concentrations, culture duration, cell seeding density, matrix composition, and barrier permeability measurements—are not fully encoded in imaging data and must be incorporated through manual or conventional quantitative analyses [138,139].

Accordingly, recent AI-enabled OoC studies increasingly adopt hybrid feature spaces that combine automatically extracted image features with manually defined descriptors and experimental metadata. Machine learning models trained on such multimodal inputs can more accurately predict biological outcomes, including tumor growth trajectories, barrier integrity changes, and therapeutic responses, than models relying on imaging data alone. In cancer-on-chip systems, for example, predictive performance is substantially improved when image-based phenotypic features are integrated with treatment conditions and functional assay results, such as viability metrics or permeability measurements [140]. Importantly, manual feature engineering remains valuable in OoC predictive modeling, particularly for capturing domain-specific knowledge. Features derived from classical analyses—such as invasion depth, growth rate constants, dose–response parameters, or transport coefficients—provide interpretable and biologically grounded inputs that complement deep learning-based representations [136]. When combined with image-derived features, these manually curated parameters enhance both model robustness and interpretability [138].

Beyond feature integration, machine learning frameworks are well suited to capture nonlinear interactions among heterogeneous inputs. Supervised learning models can associate early-stage multimodal features with downstream outcomes, enabling prediction of long-term system behavior or treatment efficacy before experimental endpoints are reached. Such predictive capabilities are especially valuable for long-term OoC experiments, where early intervention or adaptive experimental design can reduce cost and experimental burden [136]. Emerging approaches further explore hybrid predictive strategies that integrate data-driven learning with mechanistic or physics-informed constraints. By embedding prior biological knowledge or transport and growth models into machine learning pipelines, these approaches aim to improve generalizability across OoC designs and experimental conditions while maintaining predictive accuracy. In the predictive modeling of Mechano-OoC, features associated with dynamic mechanical changes should be incorporated as key elements, including the evolution curve of matrix stiffness during tumor growth and the pulse frequency of shear force. Such features can notably enhance the prediction accuracy of the model regarding tumor metastasis and drug penetration depth. For example, when stiffness gradient ranging from 1 to 10 kPa and drug IC50 value are adopted as combined inputs, the prediction error of the AI model for chemotherapy efficacy can be reduced by more than 30% [141]. Although still at an early stage, such frameworks represent an important step toward transferable and clinically relevant OoC-based prediction. 

Overall, predictive modeling in OoC systems is a multimodal process, integrating AI-derived image features, manually analyzed experimental variables, and system-level metadata [140]. This holistic approach enables OoC platforms to transition from descriptive microphysiological models toward predictive and decision-support tools, with broad implications for drug development, disease modeling, and precision medicine.

### 4.3. Challenges and Future Perspectives

Despite rapid progress, important challenges remain. AI performance depends critically on data quality, consistency, and annotation, reinforcing the need for standardized OoC protocols and comprehensive metadata reporting [137,141]. In addition, most AI models are developed for specific chip designs or experimental contexts, limiting their transferability across laboratories and platforms. Nonetheless, a consensus has emerged across extant research that artificial intelligence will constitute an indispensable, intrinsic component of Organ-on-Chip research workflows. As OoC platforms advance in maturity, and as the scale and heterogeneity of corresponding datasets expand, AI-driven image analysis, multimodal data integration, and predictive computational modeling are poised to assume increasingly central functional roles. Ultimately, the synergistic convergence of AI and OoC technologies carries considerable promise for the development of predictive, patient-centric in vitro models, which establish a critical translational interface between experimental biology and clinical decision-making. The integration of AI and Mechano-OoC, despite challenges such as data quality and model transferability, has demonstrated the potential for full-chain empowerment, covering automatic identification of mechanical phenotypes, integration of multimodal data, and prediction of therapeutic efficacy. This, together with the platform engineering and sensing technologies mentioned earlier, jointly advances the clinical translation of mechano-OoC.

## 5. Discussion

Pairing time-lapse microscopy with embedded sensors and machine learning is transforming how data is captured in Organ-on-Chip systems. Instead of relying on manual observation, these integrated setups can automatically quantify cell migration, traction forces, and barrier dynamics under cyclic strain and shear. Advanced algorithms now merge imaging streams with sensor outputs to build composite maps of mechanical stress and biochemical flux across the chip. This combined approach accelerates interpretation and enables adaptive control of experimental conditions. For instance, AI can detect early signs of endothelial barrier compromise and adjust perfusion rates or drug dosing in real time; functionality that mirrors clinical feedback systems. Such capabilities move Organ-on-Chip platforms beyond static observation toward responsive experimental ecosystems, where mechanical and biochemical signals actively guide decisions. By integrating soft electronics, microfluidics, and AI-driven analytics, these sensor-rich strategies provide a foundation for precision medicine and mechanobiology-informed drug development. Real-time fusion of AI image analysis and embedded sensor data enables dynamic correlation analysis between mechanical stimuli such as cyclic compression and cellular phenotypes such as EMT marker expression. For example, by monitoring the degree of nuclear deformation, AI can predict matrix stiffness-induced drug resistance in real time, providing a reference for the adjustment of clinical individualized treatment regimens. 

## 6. Conclusions

In summary, we have argued that the mechanical landscape of tumors, which encompasses factors from matrix stiffness and solid stress to fluid flow and confinement, profoundly influences cancer progression and therapy response. Conventional models often neglect these forces, whereas Organ-on-Chip systems offer the means to recapitulate tissue architecture, perfusion, and controlled mechanics. We emphasize that tumor-on-chip efforts must evolve to integrate mechanobiology explicitly: incorporating tunable ECM, dynamic loading, live sensing, and complex multicellular culture. Achieving this vision will require standardized platform designs and data reporting, scalable device architectures, and rigorous validation against in vivo data. By addressing these challenges, mechano-OoC could emerge as a powerful tool for studying invasion, metastasis, and treatment efficacy under realistic biophysical conditions. The mechano-OoC platforms outlined here effectively overcome the shortcomings of 2D cultures that lack mechanical cues and animal models with large species differences, through the integration of modules including tunable ECM stiffness, dynamic mechanical loading, and multicellular co-culture. Meanwhile, standardized data reporting and clinical sample verification further boost their reliability when used to replace traditional models in drug screening and mechanism-based research. By integrating four modules, engineerable tunable mechanical microenvironment, multimodal sensing, data standardization, and AI-driven interpretation, mechano-OoC not only overcomes the mechanical simulation limitations of traditional models, but also achieves the integration of mechanism elucidation and therapeutic efficacy prediction, providing a novel tool for mechanobiology-guided precision cancer therapy. The incorporation of AI technology further resolves the problems of low efficiency and insufficient predictability in the interpretation of multi-dimensional mechano-OoC data. Combined with standardized design and clinical validation, this enables mechano-OoC to become an integrated platform for mechanical mechanism research, drug screening, and therapeutic efficacy prediction.

## Data Availability

No new data were created or analyzed in this study. Data sharing is not applicable to this article.

## Abbreviations

The following abbreviations are used in this manuscript:

Mechano-OoC	Mechano-Organ-on-Chip
TME	Tumor microenvironment
ECM	Extracellular matrix
EMT	Epithelial–mesenchymal transition
OoC	Organ-on-chip
PDMS	Polydimethylsiloxane
PS	Polystyrene
PMMA	Polymethyl methacrylate
COC/COP	Cyclic olefin copolymer/polymer
GelMA	Gelatin methacrylate
LAP	Lithium phenyl-2,4,6-trimethylbenzoylphosphinate
PEGDA	Poly(ethylene glycol) diacrylate
DLP	Digital light processing
CAD	Computer-aided design
TEER	Trans-epithelial/endothelial electrical resistance
AIE	Aggregation-induced emission
DARPins	Designed ankyrin repeat proteins
COMSOL	COMSOL multiphysics
AI	Artificial intelligence
